# The COVID-19 Impact on the Trends in Yellow Fever and Lassa Fever Infections in Nigeria

**DOI:** 10.3390/idr14060091

**Published:** 2022-11-21

**Authors:** Nnennaya U. Opara, Ugochinyere I. Nwagbara, Khumbulani W. Hlongwana

**Affiliations:** 1 Institute for Academic Medicine, Department of Emergency Medicine, Charleston Area Medical Center, Charleston, WV 25304, USA; 2 Department of Health Administration, University of Phoenix, Phoenix, AZ 85040, USA; 3 Department of Public Health Medicine, College of Health Sciences, University of KwaZulu-Natal, Howard Campus, Durban 4041, South Africa; 4 Cancer and Infectious Disease Epidemiology Research Unit (CIDERU), College of Health Sciences, University of KwaZulu-Natal, Durban 4041, South Africa

**Keywords:** Lassa fever, yellow fever, COVID-19, VHFs

## Abstract

Lassa fever (LF) and yellow fever (YF) belong to a group of viral hemorrhagic fevers (VHFs). These viruses have common features and damages the organs and blood vessels; they also impair the body’s homeostasis. Some VHFs cause mild disease, while some cause severe disease and death such as in the case of Ebola or Marburg. LF virus and YF virus are two of the most recent emerging viruses in Africa, resulting in severe hemorrhagic fever in humans. Lassa fever virus is continuously on the rise both in Nigeria and neighboring countries in West Africa, with an estimate of over 500,000 cases of LF, and 5000 deaths, annually. YF virus is endemic in temperate climate regions of Africa, Central America (Guatemala, Honduras, Nicaragua, El Salvador), and South America (such as Brazil, Argentina, Peru, and Chile) with an annual estimated cases of 200,000 and 30,000 deaths globally. This review examines the impact of the COVID-19 pandemic on the trend in epidemiology of these two VHFs to delineate responses that are associated with protective or pathogenic outcomes.

## 1. Introduction

Lassa fever virus and yellow fever viruses belong to viral genera; the *Arenaviridae* and *Flaviviridae* genera, respectively. They also share similar zoological features and are often known as emerging tropical–viral diseases. Their negative impact on public health is partly due to their high virulence and human-to-human transmission (Lassa fever) and vector-to-human transmission (Yellow fever). LF and YF also share common pathogenesis in terms of their primary cell targets being myeloid cells and in terms of the organs that are affected. However, the type of immune responses that are induced by LF and YF infection and their roles in protection or pathogenesis differ significantly. These differences may play a role in understanding disease, and the differences in disease epidemiological trends during the pandemic period. This review article describes the human immune responses to LF and YF viruses and illustrates the differences between the viral spreads during the COVID-19 era. Comprehensive understanding of innate immunity to these zoonotic viruses may guide the development of effective medications that will boost human protective immune responses.

The World Health Organization (WHO) declared the coronavirus infection (COVID-19) a global pandemic on the month of March 2020. It has become one of the deadliest pandemics the world has experienced in decades. SARS-CoV-2, a virus that causes COVID-19, was first detected in Wuhan, China, in December 2019 [1]. Nigeria faces a potential public health crisis due to the synergistic epidemic of COVID-19 and other infectious diseases such as yellow fever and Lassa fever infections.

Since early 2020, the morbidity and mortality rates of COVID-19 have increased in Nigeria [2]. Simultaneously, Nigeria has recorded a high number of yellow fever and Lassa fever cases, with mosquitoes (*Aedes aegypti*) and rodents (multimammate rat) as vectors, respectively [3,4]. The spread of LF and YF has consistently been on the rise since late 2017 and early 2018 [1].

Infections with COVID-19, YF, and LF symptoms are challenging to differentiate, particularly at the early stages, due to similarities with their clinical signs. Additionally, coinfections with arboviruses or flaviviruses are not thoroughly studied in Nigeria. The country’s resource-limited healthcare system faces the risk of becoming overwhelmed with multiple socio-economic crises. A limited number of virology labs and expert virologists make it difficult to respond timely to infectious disease outbreaks and to prevent infection spread.

## 2. COVID-19 Impact on Lassa Fever Infection Rate

Lassa fever virus is an enveloped, single-stranded RNA virus, a causative agent of LF first recorded in 1969 during an outbreak in the city of Jos in Plateau state, Nigeria and was named after a city called Lassa in Borno state Nigeria [5]. LF is known to be an endemic zoonosis in some West Africa countries, especially Nigeria, Liberia, Sierra Leone, and Guinea. Current literatures have shown the disease endemicity in neighboring countries such as Mali, Ivory Coast, Togo, Cameroon, Benin, and Ghana, indicating a continuous geographical expansion of the virus and human disease [6,7]. In West Africa, the infection rate with LF annually is approximately 500,000 people, resulting in approximately 5000 deaths [8]. Also, a newly diagnosed Old World relative of the Lassa fever virus, called the Lujo virus, was identified in 2008 as the causative agent of viral hemorrhagic fever in Zambia, a country in East Africa [9].

LF is contracted through a bite from an infected rodent (*Mastomys natalensis*; a.k.a “African rat”) or via consumption of the rats, as a common practice in some endemic regions, and inhalation of virus-laden particles. Human-to-human transmission has also been seen in hospital settings [10]. The current literature on the immune responses to infection with LF has only been demonstrated in laboratory animal models, or from the in vitro culture of human cells [11].

The infections with LF are mostly asymptomatic or follow a mild course, with approximately 20% of cases resulting in moderate to severe disease with hemorrhagic symptoms and multiple organ failure [12]. The incubation period is 5 to 21 days beginning with a febrile fever, pounding headaches, diffused myalgia, and arthralgia. Patients also complain of pharyngitis with dry cough, vomiting, watery diarrhea, abdominal pain (due to hepatitis—high levels of liver enzymes, notably, the alanine amino transferase (ALT) and aspartate amino transferase (AST)) in severe cases. The poor prognosis of the disease is characterized by diffused abdominal and retrospinal pain, edema of the face and neck, lymphadenopathy, and mucosal hemorrhages. Recovery from LF generally lasts 1-3 weeks and can be associated with sensory–neural deafness. LF in pregnant women, particularly in the last trimester is often very fatal with a maternal mortality rate at 20% and fetal mortality rate near 100%. In children, LF symptoms manifest as “swollen baby” syndrome with generalized edema, abdominal distention, and mucosal bleeding [12].

Since the 1969 outbreak of LF in Nigeria, the disease has continuously occurred annually and is currently declared endemic in Nigeria, affecting over 27 states including the Federal Capital Territory. Outbreaks of LF mostly occur during the harmattan or dry season in Nigeria with the peak incidence occurring between October-April [13]. In 2020, Nigeria recorded 6791 suspected cases of LF with 1189 confirmed cases and 244 deaths. The disease affected 27 states with a case fatality ratio of 19.3%, less than the 20.9% reported in 2019 [14].

**Figure 1:**
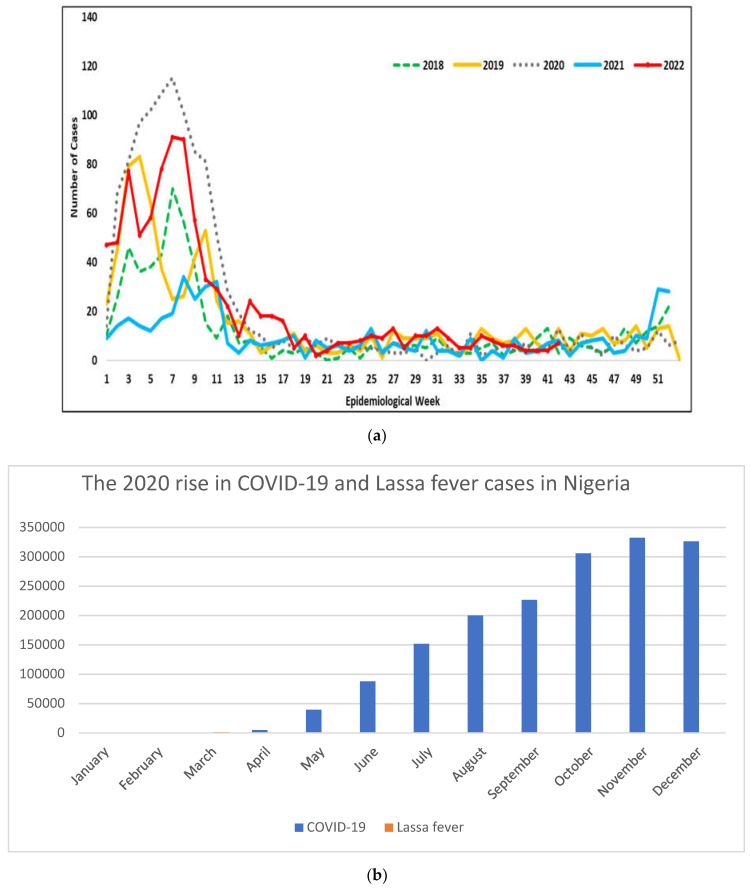

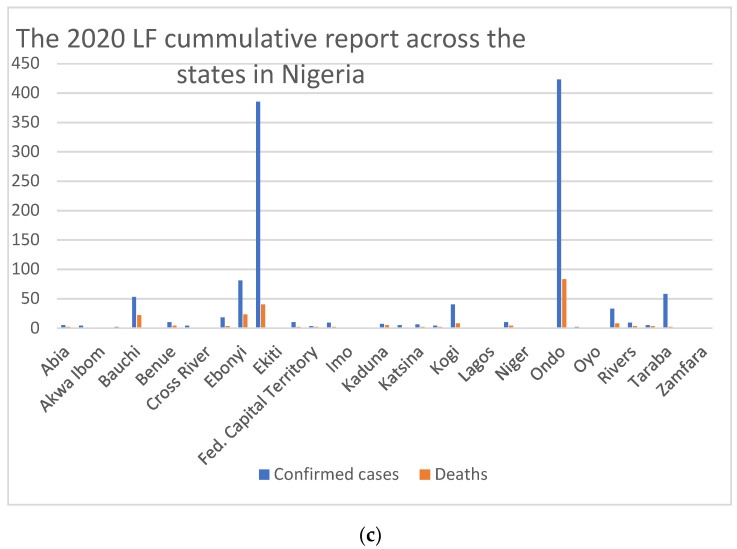
(**a**) Trend in confirmed cases of LF by epidemiological week from year 2018 to 2022. The COVID-19 pandemic era showed the highest number of LF cases recorded compared to previous years. The data also show a high increase in new cases of LF in the current year (2022) during the harmattan season (dry season) as the Nigerian Center for Disease Control (NCDC) continues to see a concurrent rise in new cases of COVID-19 infections in hospitals. In 2021, LF cases plummeted as large number of people became vaccinated against COVID and due to continuous implementation of COVID-19 infection control measures. (**b**) Shows the surge in COVID-19 infection with a concurrent rise in Lassa fever in 2020. The COVID-19 infection rate was high across the 36 states in the country, outnumbering the cases of LF even. Data obtained and reprinted with permission from the Nigerian Center for Disease Control (NCDC) (copywrite 2022) [15]. (**c**) The 2020 Lassa fever epidemiology cases across the states in Nigeria. Data obtained from the Nigerian Center for Disease Control [15].

We compared weekly surveillance activity for Lassa fever in the 2020 season with activity in four previous years using data from 2018–2022 from the National Lassa fever Emergency Operations Center [Figure 1a,b]. The center gathers the total number of newly confirmed weekly cases of Lassa fever based on clinical symptoms and laboratory findings from 44 centers.

There are similarities in the epidemiological trend of LF from 2016 to 2022. However, the highest peak in infection with LF was seen during the COVID-19 pandemic compared to other years of LF infection peak seasons in Nigeria. This could be explained by the impact of the SARS-CoV-2 infection on the human immune response to other viral infections including the VHFs.

The main target cells of the LF virus are the myeloid cells, which include macrophages and dendritic cells. The LF virus arrests dendritic cells (DC) in an immature, inactive state despite DC migration to the lymph nodes, and the greater disruption in the function of antigen-presenting cells (APCs) by LF viruses compared to SARS-CoV-2 might correlate with the general lack of adaptive immune responses in fatal LF. It is also important to note that the scientific findings regarding the interactions between LF and SAR-CoV-2 viruses and myeloid cells were made using human cells extracted and derived in cell culture, which does not accurately depict all features of infection and cell maturation *in vivo.* The human innate immune response upon initial sensing of virus infection produces interferons (IFNs) for macrophages and DCs to increase the activities of co-stimulatory molecules that are required for antigen presentation and for enhancing T-cell responses. The importance of these natural immune responses in viral attack is supported by several mechanisms that are encoded by vertebrate viruses to elude these responses [16], for example, the LF virus blocks the actions of IFN by warding off viral RNA sensing, which prevents the maturation of infected DCs. The small protein Z matrix of LF virus inhibits signaling via both retinoic acid-inducible gene (RIG-1) and melanoma differentiation-associated protein 5 (MDA5) by binding to the caspase-recruitment domain (CARD) of the two protein molecules, blocking its interaction with the downstream adaptor mitochondrial antiviral-signaling protein (MAVS) [17]. Additionally, Lassa fever viral nucleoprotein utilizes two strategies to suppress the actions of Type 1 IFN. First, the viral nucleoprotein encodes an exonuclease activity that has strong specificity for viral dsRNA, resulting in its breakdown [18,19]. Such activity supposedly destroys viral RNA that is not directly involved in replication or transcription and translation and prevents its recognition by RIG-1-like receptor (RLR). Second, nucleoproteins of the LF virus bind to 1kB kinase (IKK) and inhibit its activation of the downstream transcription factors interferon regulatory factor 3 (IRF3) and nuclear factor (NF-kB) [20,21]. Similarly, SARS-CoV-2 blocks the activation of Type 1 and Type III IFN responses by their non-structural proteins, which affect the production of cytokines [22]. These combined effects of the SARS-CoV-2 and LF virus on the human immune system, and subsequent human immune response to the virus, could explain the trend in LF infection during the peak of the COVID-19 pandemic period compared to previous years [Figure 1a–c].

On the other hand, sRNA derived from SARS-CoV-2 genomic can be detected by RIG-1 and MDA5 intracellularly. SARS-CoV-2 has evolved several evasion strategies to counteract the human innate immune response (induction of inflammatory responses that reduces viral replication) by decreasing the levels of IFNs; patients with mild and moderate COVID-19 have low levels of Type 1 and Type 3 IFNs in their blood serum [23]. Evidently, SARS-CoV-2 infection decreases the production of IFNs I and III at post-transcriptional points by interfering in the flow of mRNA from sites of transcription, or by stimulating transcription breakdown in the nucleus. These innate immune system destabilization and evasion strategies by SARS-CoV-2 are believed to pave a way for other viral infections [24]

Management of patients diagnosed with LF could be challenging considering the disease’s insidious onset and non-specific clinical symptoms. However, supportive and symptomatic treatment with intravenous fluid resuscitation and electrolyte balance is beneficial in LF patients. An antiviral drug (Ribavirin) has proven successful in the early treatment of patients diagnosed with Lassa fever LF.

## 3. COVID-19 Impact on Yellow Fever Infection Rate

Yellow fever (YF) is an acute viral hemorrhagic infectious disease caused by the YF virus, an RNA virus from the Flavivirus genus, and transmitted by bites from mosquitoes belonging to the *Aedes* and *Haemogogus* species [25]. The three common transmission cycles are:

- Sylvan YF: also known as “jungle” yellow fever infestation, this occurs in the temperate climate regions (tropical rainforests commonly seen in Africa and South America) where the primary carriers of the virus are monkeys following bites by wild mosquitoes of the *Aedes* and *Haemogogus* species. Humans become infected when they tour or work in the rainforest and are bitten by infected mosquitoes [25].
- Intermediate YF: here, the semi-domestic mosquitoes (wild and household bred) infect monkeys and humans. Increased contact between people and infected mosquitoes leads to increased transmission, especially in densely populated regions. this type of transmission is the most typical type of outbreak in Africa [26].
- Urban YF: this is slightly like the intermediate type, as transmission occurs when the infected group of people spread the YF virus in heavily populated regions with several breeding grounds for *Aedes aegypti* mosquitoes and where people with no immunity due to lack of vaccination live. In this condition, infected mosquitoes transmit the virus from person to person [26].

Yellow fever is distributed in the west, central, and east Africa, Central America, and South America [27]. YF remains a public health emergency despite the availability of a safe and effective vaccine, with an estimated mortality rate of 30,000 and an incident rate of 200,000 annually [27].

YF symptoms can take 3–6 days to develop and include fever, chills, headache, backache, and muscle aches. About 15% of patients diagnosed with YF will develop jaundice, conjunctival hemorrhage, epistaxis, hematochezia, coffee ground emesis, hemorrhagic shock, organ failure (most commonly liver and kidney), and death [28]. Severe YF infection can be deadly, with a mortality rate between 30–60%. The new infections in humans spread through aerosol mode or air droplets mode of transmission (saliva from an infected person to a non-immune person) [28].

The disease is endemic to sub-Saharan African and South American countries with temperate climates. The first case of YF was not documented; however, the first incident of a presumed case of YF was eventually documented in a Mayan article and described as hematemesis in Yucatan, Mexico, in 1648 [29]. The YF virus and its vector (*Aedes* mosquitoes) were believed to have been introduced into Mexico by slaves from endemic countries in West Africa during the slave trade period. Additionally, around the time of the slave trade with the migration of slaves to the United States, an epidemic of YF was reported in several cities, with a 10% mortality rate from YF recorded in Philadelphia in the year 1793 [29]. The first outbreak of YF in Nigeria was reported in the city of Lagos in 1864 [30].

YF cases have been on the increase over the past few years, with each year’s outbreak surpassing the previous year. However, the outbreak of COVID-19 in 2020 was different. Despite the introduction of the YF vaccine into the 2004 regular immunization schedule of Nigeria, the incident rates of YF have continuously been on the rise with suboptimal mass immunization and rising rates of urbanization within the neighboring African countries, particularly Nigeria [30,31]. As of 2018, Nigeria has one-third of the population at risk of yellow fever, with approximately 112 million people remaining unvaccinated against YF [32].

**Figure 2:**
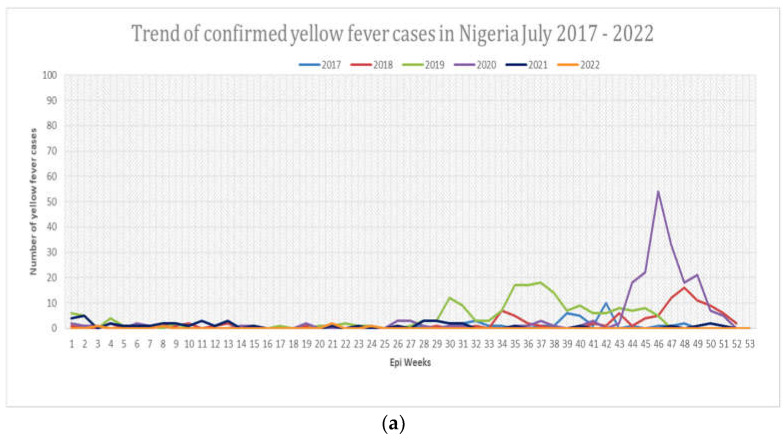

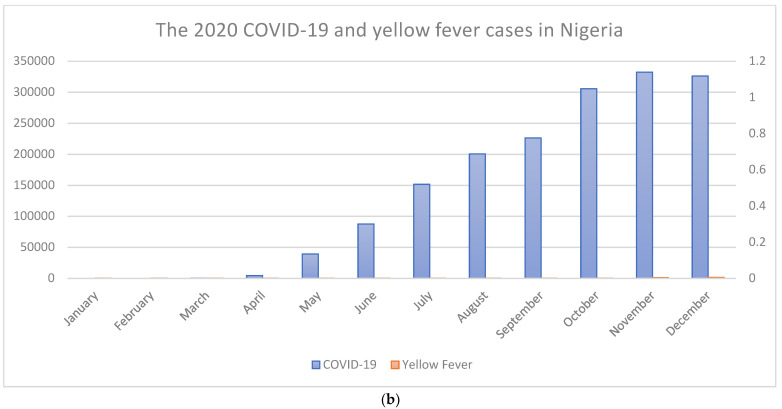
(**a**): Comparing epidemiological spread of yellow fever in 2017–2022. The 2020 had the highest number of confirmed cases of YF than any other years in Nigeria. The common season for most reported cases of YF is the months of September through December. In 2021, there were few cases of YF as most people became vaccinated, and due to continuous implementation of COVID-19 prevention protocols. However, as COVID-19 restrictions become completely phased out, and hospitals across Nigeria began to see rises in COVID-19 new cases, infections with YF begins to rise higher than pre-COVID-19 years, as seen in Figure 2. This further suggests a possible correlation between SARS-CoV-2 and yellow fever co-infection. (**b**) Shows consistent rise in COVID-19 infection with a concurrent rise in yellow fever infection, even though not visible on the chart base on the higher number of COVID-19 cases compared to yellow fever cases.

In Nigeria, YF surveillance is case-based weekly surveillance with documentation on the probable cases and confirmed cases reported to health authorities at the local government areas (LGAs), as well as at state and national levels [Figure 2].

The E protein, a heavily mutated region of the entire genome plays a key role in viral entry into immune cells. The E protein serves as the main target for the YF-17D vaccine by causing a mutation in the E protein thus, altering the YF virus tropism and affecting its virulence [33,34,35,36]. The peak season for YF virus infection in Nigeria is usually during the dry season between September–December. It is believed that coinfections with COVID-19 and YF may have played a significant role in the decrease or increasing disease outbreaks and severity [37]. When immune cells are coinfected by two viruses of the same structure (RNA-RNA) a virus usually affects the duplication or replication of the other virus, a process known as viral interference, which often results in the clearance or removal of one virus with the existence of the other [38]. Additionally, viral interference could be induced by several other factors including interferon suppression, production of E proteins (as in the case of YF), cellular T cell activations, and non-specific double-stranded RNA (dsRNA) [39]. it is important to note that both SARS-CoV-2 (main target cell Angiotensin Converting Enzyme-2 (ACE2)), the YF virus, and other CoVs collectively limit the activities of the cell surface, endosomal, and cytosolic pattern recognition receptor (PRRs) responses to pathogen-associated molecular patterns (PAMPs) that trigger inflammatory responses and programmed cell death, which limits viral infections and clearances, thus enabling the possibility of viral coinfection [40].

The human immune response also affects the development of viral coinfections. This can be further explained by the actions of the naïve T cells upon sensing a viral infection, which convert into activated T cells and later into memory T cells. The memory T cell responses, which are generated to act against a particular viral infection, could influence the amount and strength of the immune response to any future or unrelated viral coinfection, a process called heterologous immunity (HI). The HI can be seen between viruses of similar structure or genus, multiple variants of the same virus type, among different viruses, between virus and bacteria, or between virus and protozoa [41]. Therefore, we hypothesize that COVID-19 infection potentiated the virulence of YF resulting in higher cases and deaths during the peak season (dry season) in the COVID-19 era.

In week 1 to 53 of the year 2020 (Figure 2a,b), the Nigeria Center for Disease Control reported 3426 suspected cases of Yellow Fever. Of the reported suspected cases, 145 were confirmed, with 17 deaths from the documented cases [14] (Figure 2a,b). It was hypothesized that the reasons people presented to the hospital with YF during the COVID-19 pandemic in the later months of 2020 were either due to severe symptoms of YF (ocular jaundice, abdominal pain, pruritus, and bleeding), or due to increased demand and decreased supplies of long-lasting insecticidal nets (LLINs) by the residents in the densely populated areas, caused by nationwide lockdown, resulting in increased exposures to mosquito bites.

Diagnosing yellow fever during an outbreak of a pandemic (COVID-19) can be very challenging as it is clinically difficult to distinguish yellow fever from other infectious diseases (hepatitis A, B, or C) and COVID-19 based on clinical symptoms, especially when presenting with mild or atypical symptoms [14]. Serological testing with polymerase chain reaction (PCR) in blood and urine samples could detect the virus in its early stages [14]. The enzyme-linked immunosorbent assay (ELISA) to see yellow fever viral IgM antibodies provides an early diagnosis of YF.

There is no definitive treatment for YF, only symptomatic management. Vaccination against the yellow fever virus provides life-long immunity.

## 4. Public Health Interventions Necessary for Reducing the Spread of LF and YF Viruses

All infectious diseases require a specific set of health interventions in the reduction of their transmission, morbidity, and mortality and their impact on the health system at large. A study showed that the most effective measure for decreasing the spread of Lassa fever is an increase in the death rate of the rodents by mice culling which alternatively decreases the number of infected rodents (African rats), and even could drive the disease to complete extinction [42]. An additional measure is to reduce human-to-human transmission rates by increasing personal hygiene such as frequent handwashing, and the use of personal protective equipment (PPE) when caring for infected persons in the case of LF. Decreasing rodent-to-human transmission by using rodent-safe food containers and collecting garbage far from houses can assist in eradicating the disease [42].

In the case of YF, an emergency mass vaccination with the YF-17D vaccine and vector control are needed in the control of yellow fever as the mass vaccination will help in increasing the population immunity and survivability rates in the country [43]. Community engagement is essential in the elimination of breeding sites of the *Aedes* mosquitoes and preventing them from growing from the egg to larva and adult, by educating the public on the proper use of long-lasting insecticidal nets (LLINs) and ensuring that there is no breech in the supply of LLINs in the endemic areas [43].

## 5. Conclusions

Nigeria’s COVID-19 response represents a unique situation of effectively implementing population-wide infectious disease control measures such as mask-wearing, isolation of infected people, personal protection, practicing good hygiene, and immunization. Additionally, a strong knowledge of the determinants of viral coinfections and interactions between two viruses remain as an emerging field in virology research. It is important to note that laboratory testing of clinical specimens often produces false-positive results when we try to link such results with a single identified pathogen as a cause of a disease process. Frequently, additional pathogens that contribute to the worsening of a disease outcome are undetected. As a result of viral interference as described in the article, one virus may lead to either genetic recombination or genetic reassortment, which subsequently yields antigenic variants that can be missed by passive immunity (vaccine-induced immunity) and antiviral drugs as in the case of yellow fever outbreaks in the dry season seen in Figure 2, and Lassa fever outbreak in Figure 1a,b.

It is also imperative to note that a strong acquired immunity is crucial for the efficient control of pathogens involved in heterologous infections. So, memory T cells’ responses to an individual virus can significantly affect both the amount and quality of immune response available against subsequent viral infections or any type of infection. Additionally, it is important to note that the subsequent infections may either decrease the amount of existing immune memory cells or produce additional effects that could boost immune defenses.

In summary, based on the LF and YF disease patterns during the COVID-19 era (2020) we can strongly agree that there was indeed a correlation in the trends of YF and LF during their individual peak seasons (dry seasons), and with the continuous infection of the COVID-19 pandemic, the outcomes of viral coinfections need to be thoroughly explored and clearly comprehended in addition to developing advanced methods for diagnostics, preventive vaccines, and antiviral medications.

## Data Availability

The data used in this study are from the Nigerian Center for Disease control.
